# Food Insecurity among People Living with HIV/AIDS on ART Follower at Public Hospitals of Western Ethiopia

**DOI:** 10.1155/2020/8825453

**Published:** 2020-07-25

**Authors:** Adugna Oluma, Muktar Abadiga, Getu Mosisa, Werku Etafa, Ginenus Fekadu

**Affiliations:** ^1^School of Nursing and Midwifery, Institute of Health Sciences, Wollega University, Nekemte, Ethiopia; ^2^Department of Pharmacy, Institute of Health Sciences, Wollega University, Nekemte, Ethiopia

## Abstract

**Background:**

Food insecurity and HIV/AIDS are intertwined in a vicious cycle through nutritional, mental health, and behavioral pathways. Food insecurity is a potentially important barrier to the success of antiretroviral treatment, increased hospitalizations, and higher morbidity among HIV-infected individuals in resource-poor settings particularly in sub-Saharan Africa including Ethiopia. Therefore, the purpose of this study was to assess the prevalence of food insecurity among people living with HIV/AIDS on follow up at public hospitals of western Ethiopia.

**Methods:**

An institutional-based cross-sectional study design was conducted on a sample of 428 among people living with HIV/AIDS on follow up at public hospitals of western Ethiopia. A systematic random sampling technique was used to include all participants. Data was collected using interviewer-administered structured questionnaires. The data were entered into Epi data version 3.1 and then exported into Statistical Package for the Social Sciences window version 21 for analysis. Descriptive and inferential statistics were employed. Bivariable logistic and multivariable logistic analyses were used with AOR at CI 95% and *p* < 0.05 were used.

**Result:**

The finding of the study revealed that the majority of the respondents 221(53.1%) were female. The mean age of the respondents was 32.92 ± 7.304 years and 197 (47.4%) of the study participants were between 30 to 39 years' age group. The level of food insecurity among PLWHA receiving ART therapy was 68.8% which was partitioned as mild (23.32%), moderate (29.09%), and severe (16.35%) food in secured. Being single [AOR = 3.507(1.377, 8.934)], illiterate [AOR = 5.234(1.747, 15.686)], cigarette smoking [AOR = 3.577(2.104, 6.081)], presence of anemia (AOR = 2.650(1.563, 4.493)], and inadequate dietary diversity [AOR = 2.870(1.088, 7.569)] were predictors of food insecurity.

**Conclusion:**

The prevalence of food insecurity was high. Educational status, marital status, cigarette smoking, presence of anemia, opportunistic infection, and inadequate dietary diversity were the major significant factors affecting food insecurity. We recommended a national health policy maker to integrate food and nutrition interventions as part of a package of care, treatment, and support services for people living with HIV and ART follower patients.

## 1. Introduction

It is estimated that over 2 billion people worldwide are affected by food insecurity that is linked to the HIV epidemic in both resource-rich and resource-poor settings. The Food and Agriculture Organization of the United Nations (FAO) reports the burden of food insecurity faced the world population was about 26.4% experienced the combination of moderate and severe levels of food insecurity together with those suffered from hunger [1].

The global prevalence of undernourishment was widely inconsistent with 10.8% worldwide, 19.9% Africa, 11.3% Asia, 6.5% Latin America and the Caribbean, 6.2% Oceania, and <2.5% northern America and Europe. Approximately, 89% of food-insecure individuals live in Asia and Africa. About 28.5 million HIV/AIDS infected people live in sub-Saharan Africa. About 70% of them were food insecurity and malnutrition [2, 3].

The four-tier dimensions of food security are availability of food, access to food, utilization of food, and stability. Food insecurity refers to the unavailability of adequate and sustainable food supply, inability to access adequate balanced diet, and inability to utilize safe and quality food which is nutritionally adequate and socially acceptable ways for all household members [4].

The Human Immune virus/Acquired Immunodeficiency syndrome (HIV/AIDS) and food insecurity have a bidirectional relationship. Food insecurity increases the risk of HIV infection and cause poor nutritional status. HIV infection causes food insecurity by reducing agricultural production, reducing income, and increasing a medical expense that leads to reduced capacity to respond to the crisis [5].

HIV/AIDS and food insecurity have a cyclical relationship. HIV/AIDS affects food insecurity mainly through its corrosive effects on people's economic sustainability, loss of income as a result of disease progression. Food insecurity is recognized as a key determinant of reduced adherence to antiretroviral therapy, increased behavioral risk of HIV transmission, reduced access to HIV treatment, adverse antiretroviral pharmacokinetics, and worse clinical outcomes among HIV infected individuals [6, 7].

Food insecurity and poor nutritional status causes lower CD4 cell counts, poor adherence to ART, decreased viral suppression, and increase morbidity and mortality. It can also accelerate the development of opportunistic infections and result in the progress of AIDS-related illnesses among People Living with HIV AIDS [8].

The study showed that food insecurity can lead to macronutrient and micronutrient deficiencies. These deficiencies affect the vertical and horizontal transmission of HIV that contributes to reduced immunity and leads to an increase in morbidity and mortality. It can have mental health consequences, including depression, increase drug abuse, accelerate HIV transmission, incomplete viral load suppression, and increased probability of AIDS-defining illness among HIV-infected persons [9].

Worldwide, one-third of 40 million PLWHA are coinfected with opportunistic infections related to malnutrition that weakens the immune system, leading to greater susceptibility. About 41% of adults and 32% of children had access to antiretroviral treatment [10].

Studies conducted in various countries among PLWHA receiving HAART showed a high prevalence of insecurity indicated that Canada 71%, Democratic Republic of Congo, 57%, Namibia 92%, and Kenya revealed that 20-50% were food insecure [11–14].

HIV/AIDS and food insecurity are two of the leading causes of morbidity and mortality that increase vulnerability and worsening the severity of one another. The prevalence of food insecurity among PLWHA was high in sub-Saharan Africa, particularly in Ethiopia. The figures are Ethiopia 63%, Uganda 75%, and Tanzania 52% [6, 15, 16].

In Ethiopia, about 1.5% of adult people aged 15-49 are infected with HIV that was intensely affected by food insecurity. It was estimated that almost 1 in 10 Ethiopians struggled to have access to safe, sufficient, and nutritious food for their families [17].

Study showed that lower age, lower educational level, marital status, low health status, political, and demographic factors such as gender, income, body mass index, smoking, household composition, nutritional status, physical and mental development, social vulnerability to infectious, and chronic diseases are related to food insecurity [18].

Common factors influencing food insecurity are household's ability to pay for food, physical access to adequate food, health requirements for nutritious food, preferences for culturally appropriate food, income, the costs of food, geographic isolation, lack of transportation, and food literacy. High-risk populations are lone-parent families, women, and children; immigrants and elders are more likely affected by key risk factors [19].

Despite of the fact that food insecurity compromises the effectiveness of HIV treatment, reduce ART adherence, and induce HIV related stigma, isolation and anxiety understanding the predictors of food insecurity is crucial to create awareness for social support for infected patients and integrates comprehensive nutritional therapy for infected individuals [20]. The status of food insecurity is not well known among PLWHA in West Ethiopia. Therefore, this study was designed to fill this gap and determine the levels and predictors of food insecurity among adult patients on follow up at public hospitals of Western Ethiopia.

## 2. Materials and Methods

### 2.1. Study Setting and Population

The study was conducted in four Public Hospitals of western Ethiopia including Nekemte referral Hospital, Ghimbi hospital, Jimma Arjo Hospital, and Nedjo hospital from September 2019–October 2019. The hospitals were randomly selected by the lottery method from all hospitals found in western Ethiopia. An Institutional based cross-sectional study design was employed. The sampling frame was the list of all recorded people living with human immune deficiency virus on patient medical records at each ART clinic of the respective hospitals which was 5208. All PLWHA on follow up receiving antiretroviral therapy at the selected hospitals were the source population. The study populations were all of the people living with human immune deficiency virus on follow up actively receiving antiretroviral therapy at each ART clinic of respective hospitals. The study subjects were all sampled people living with human immune deficiency virus actively on follow up receiving antiretroviral therapy and presented during the data collection period. All PLWHA above 18 years and receiving ART more than 12 months were included. PLWHA did not present on follow up during the study period were excluded from the study.

### 2.2. Sample Size Determination and Sampling Techniques

The sample size of the study was calculated using the formula for estimation of a single population proportion with the assumptions of 95% Confidence Level (CL), marginal error (d) of 0.05, and proportion of 0.63 (63%) taken from the previous study conducted in Jimma university specialized hospital [21]. The respondents of the study were included by a systematic random sampling technique. The total sample size was included systematically by calculating constant *k* value from all PLWHA on follow up actively receiving antiretroviral therapy which was twelve. The initial number was selected randomly from the interval of one to twelve which was seven picked by balloting. Based on proportionate allocation participants were included every twelve intervals. By adding, 10% a nonresponse rate the total number of participants recruited for the study was 428.

### 2.3. Data Collection Tool and Procedures

Data were collected using an interviewer-administered questionnaire. Data collection tools consist of three-part questionnaires. The first parts were sociodemographic related questionnaires developed by investigators. The second part was the House Hold Food Access Scale (HFIAS) were taken from the previous study [22] which was originally developed by Food and Nutrition Technical Assistance (FANTA) that measures food insecurity in terms of 18 questions items. The 18 question items have two components. The first 9 items are occurrence questions responded in terms of yes = 1 or no = 0 which has minimum value 0 to maximum value 9. The second 9 items are frequency questions derived from occurrence questions with the response option of yes. The frequency questions have an option of rarely = 1 (once or twice in the past four weeks.), sometimes (three to ten times in the past four weeks.) =2, and often (more than ten times in the past four weeks) =3, which has a minimum value of 9 to maximum value of 27. The overall of Cronbach's alpha the tool was 0.938.

The third part was the dietary Diversity Index taken from tools originally developed by the Food and Agriculture Organization (FAO) [23] with a total 7 food items that measure household food insecurity in terms of varieties of nutrients eaten by households within the previous twenty-four hours. The seven food group items have two possible values: 1 = yes (if the household/individual consumed that specific food group within the previous twenty-four hours) and 0 = no (if they did not consume that food within the previous twenty-four hours), which has a minimum value of 0 to maximum value of 7. The overall of Cronbach's alpha the tool was 0.644. A Close-ended interviewer-administered structured questionnaire was distributed to participants by trained data collectors. The data collectors were qualified and experienced previously had an exposure of data collection working in different hospitals (Additional File 1).

### 2.4. Data Control and Management

All questionnaires were translated into the local language Afan Oromo and then translated back into English languages by experts. A pretest was conducted on 5% of the sample size at shamboo hospital that was outside the actual study setting before data collection. The one-day training was also given for data collectors and supervisors. Data were cleaned, coded, and checked for consistency and completeness. The principal investigator prepared the template and entered data using Epi Data version 3.1. Finally, after missing, value and incorrect entry checked the data was exported to SPSS version 21.

### 2.5. Data Processing and Analysis

Data was cleaned, edited, coded, and entered into Epi data version 3.1 and was exported to SPSS windows version 21 for analysis. Descriptive statistics including percentage, proportion, and frequency table was used to describe the data. A chi-squared test was employed to determine factors associated with food insecurity. Binary logistic regression was used for multivariable analysis. All variables significant at *p* value < 0.25 in the bivariable logistic regression were entered in multivariable logistic regression analysis. A backward stepwise model was used to include all variables that are suitable in multivariable logistic regression analysis. The goodness fit was fixed by Hosmer and Lemeshow test. Finally, multivariable logistic regression analysis with AOR, CI at 95%, and the significance level was set at *p* < 0.05. Finally, the results of the study were reported in terms of by tables, figures, and narrated paragraphs.

### 2.6. Operational Definition

#### 2.6.1. Food Security

Means when all respondents say “no” for all affirmative household food access scale of occurrence questions measured in terms of 9 items for at least four weeks (4) duration.

#### 2.6.2. Food Insecure

Individuals were labeled to be food insecure if they answer “Yes” to all affirmative household food access scale of occurrence questions measured in terms of 9 items for at least four weeks (4) duration. This can be labeled as mild, moderate, and severe food insecurity tertian classification method.

#### 2.6.3. Mild Food Insecurity

When all respondents responded rarely (1) for frequency questions with a value interval between 1-9 inclusively.

#### 2.6.4. Moderate Food Insecurity

When all respondents responded sometimes (2) for frequency questions with a value interval between 10-18 inclusively.

#### 2.6.5. Severe Food Insecurity

When all respondents responded often (3) for frequency questions with a value of 27 (3X9 = 27).

#### 2.6.6. Food Availability

Means when the respondents say food is physically present in sufficient quantities for at least six (6) month duration.

#### 2.6.7. Irrigation

A household's artificially supplying and systematically dividing of water for agriculture in order to ensure food security.

#### 2.6.8. Farmland

A household's agricultural land for crop production.

## 3. Result

### 3.1. Sociodemographic Characteristics of Respondents

Four hundred and sixteen respondents participated in the study giving a response rate of 97.2%. The majority of the respondents 221(53.1%) were females. With regard to marital status, the highest proportion of 260 (62.5%) were married. The mean age of the respondents was 32.92 ± 7.304 years. About 197 (47.4%) of the study participants were aged between 30 to 39 years' age group. With regards to educational status, about 114 (27.4%) of the respondents were illiterate followed by those who could read and write 106(25.5%). Concerning their occupation, the majority of the respondents 144(34.6%) were daily laborers. The study also showed that the majority of respondents 238(57.2%) gain income less than 600 EBR. Moreover, more than half 233(56.0%) of the respondents were urban dwellers (Table 1).

### 3.2. Clinical Profiles, Nutritional, and Art Status among the Respondents

More than half 245 (58.9%) of the study participants were at clinical WHO stage I and nearly quarter 102 (24.5%) were at clinical WHO stage III. The mean and standard deviation of the body mass index of the respondents was 18.60 ± 3.612. About one-third of 136 (32.7%) of the study participants had CD4 count between 351-500 cells/ul. Around 64 (15.4%) of the respondents had Pneumocystis Carinii pneumonia. With regards to the ART regimen, nearly half of the respondents had taken 202 (48.6%) TDF + 3TC + EFV therapy during follow-up. The majority of the respondents 216 (51.9%) were anemic as well as they were cigarette smokers 254 (61.1%). With regards to nutritional counseling, 353 (84.9%) had dietary counseling. About 391 (94.0%) of the respondents had inadequate dietary diversity (Table 2).

### 3.3. Prevalence of Food Insecurity among the Respondents

**Figure 1:**
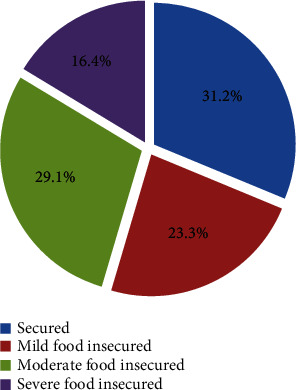
Pie chart illustrating the level of food insecurity among the respondents among people living with HIV/AIDS on follow up at public hospitals of western Ethiopia.

The overall proportion of food insecurity among PLWHA receiving ART at therapy at public hospitals of western Ethiopia was 68.8%. The level of food insecurity among participants classified as mild (23.3%), moderate (29.1%), and severe (16.4%) food insecurity (Figure 1).

### 3.4. Bivariable Logistic Regression Analysis Result of Factors Associated with Food Insecurity among the Respondents

All variables significant at *p* value < 0.25 in the bivariable logistic regression were qualified for multivariable logistic regression analysis. Among significant variables were sex, marital status, educational status, occupation, and place of residence, family size, and stage of HIV/AIDS, anemia, farmland, and presence of opportunistic infection, cigarette smoking, and dietary diversity were significant variables with food insecurity (Table 3).

### 3.5. Multivariable Logistic Regression Analysis Result of Factors Associated with Food Insecurity

All variables significant at bivariate logistic regression entered into a multivariable regression model. Six variables significantly associated with food insecurity among PLWHA on follow up receiving ART in the multivariable analysis were marital status, educational status, presence of opportunistic infection, anemic status, cigarette smoking, and dietary diversity. Being illiterate was 5.234 times more likely to be food insecure when compared with those who were completed college/university (AOR = 5.234, 95% CI: 1.747, 15.686). With regards to marital status, those who are single were 3.507 times more likely to be food insecure when compared with those who were widowed (AOR = 3.507, 95% CI: 1.377, 8.934). Concerning smoking status, those who were smokers were 3.577 times more likely food insecure compared with those who were nonsmokers (AOR = 3.577, 95%CI = 2.104, 6.081). With regards to anemia status, those who were anemic were 2.650 times more likely food insecure compared with those who were not anemic (AOR = 2.650, 95%CI = 1.563, 4.493). Concerning dietary diversity, those who had inadequate dietary diversity were 2.870 times more likely food insecure compared with those who had adequate dietary diversity (AOR = 2.870, 95%CI = 1.088, 7.569) (Table 4).

## 4. Discussions

The aim of this study was to assess the level and factors associated with food insecurity among people living with HIV/AIDS on ART follower sat selected west Ethiopia public hospitals. The result of the study showed that the prevalence of food insecurity was 68.8%. This finding was relatively higher than the result of studies conducted in Arba Minch General Hospital, Southern Region (19.5%), Kenya (20-50%), Uganda (37.9%), Democratic Republic of the Congo (57%), Namibia (67%), Humera Hospital in Northern Ethiopia (40.4%), Jimma University Referral Hospital (63%), and Butajira hospital southern nation nationalities and peoples (67.5%) but lower than the result of the study conducted in Fiche Zonal Hospital in Oromia region (87.4%). The variation among different parts of the countries could be due to the existence of different socioeconomic status, the health intervention measurement taken, and difference in study years and study setting. In addition due to variation in tools used to measure food insecurity [10, 12–15, 24–26].

The study revealed that educational status was the strongest factor independently associated with food insecurity considering the joint effect of other variables in the regression model. Being illiterate was 5.234 times more likely to be food insecure when compared with those who were completed college/university. This finding was similar to a study conducted in Jimma university specialized hospital that revealed respondents who completed elementary or less were 3.10 times more likely to be food insecure when compared with those who completed secondary and above. In a similar way, a study conducted in Portugal indicated having no education was 7.98 times more likely to be food insecure when compared with completed greater than 10th grades. This similarity justifies the fact that improvement in the educational status improves the living standard of the total community [15, 27].

The study also indicated that cigarette smoking was a factor independently associated with food insecurity considering the joint effect of other variables in the regression model. Respondents who were cigarette smokers were 3.577 times more likely food insecure compared with those who were nonsmokers. This finding was similar to a study conducted in the Portuguese population that revealed respondents who were cigarette smokers were 1.56 times more likely food insecure compared with those who were nonsmokers. This similarity might be related to the fact that cigarette smoking decreases the individual income and expose to additional extravagancy through expending money for paying tobacco packets per day [27].

The finding of the study also showed that respondents who developed opportunistic infection were 3.108 times more likely food insecure compared with those who did not develop opportunistic infections. This finding was similar with the finding of a study conducted in Arba Minch General Hospital southern nation nationalities and peoples of Ethiopia showed respondents who developed opportunistic infection were 8.03 times more likely food insecure compared with those who did not develop opportunistic infections. The possible justification might relate to the fact that the presence of opportunistic infections leads to decreased food utilizations and stability that contribute to food insecurity [24].

The study also showed that the presence of inadequate dietary diversity was strongly associated with food insecurity. Participants who did not have adequate dietary diversity were 2.870 times more likely food insecure compared with those who had adequate dietary diversity. This finding of the study was similar to conducted in Butajira hospital southern nation nationalities and peoples of Ethiopia that revealed participants who did not have adequate dietary diversity were 14.1 times more likely food in secured compared with those respondents who had adequate dietary diversity. This similarity justifies the fact that lack of ingestion of a variety of nutrients leads scarcity of essential micronutrients that contribute to the development of food insecurity [19].

### 4.1. Limitation of the Study

Causality cannot be confirmed since the research design is cross-sectional.

## 5. Conclusion

The proportion of food insecurity among PLWHA on follow up receiving ART was found relatively high. This study found that educational status, marital status, cigarette smoking, presence of anemia, and inadequate dietary diversity were positively related to food insecurity. Enhancing educational status and behavioral modification is essential to improve food security. We recommended a national health policy maker to integrate food and nutrition interventions as part of a package of care, treatment, and support services for people living with HIV and ART follower patients.

## Figures and Tables

**Table 1:** Sociodemographic characteristics of respondents among people living with HIV/AIDS on follow up at public hospitals of western, Ethiopia, 2019 (*N* = 416).

Variables	Category	Frequency (%)
Sex	Male	195 (46.9%)
	Female	221 (53.1%)
	Total	416

Age	≤19	16 (3.8%)
	20-29	115 (27.6%)
	30-39	197 (47.4%)
	≥40	88 (21.2%)
	Total	416

Marital status	Married	260 (62.5%)
	Single	61 (14.7%)
	Divorced	28 (6.7%)
	Widowed	67 (16.1%)
	Total	416

Family size	≤3	236 (56.7%)
	4-5	126 (30.3%)
	>5	54 (13.0%)
	Total	416

Educational status	Illiterate	114 (27.4%)
	Can read and write	106 (25.5%)
	Completed primary	81 (19.5%)
	Completed secondary	74 (17.8%)
	Completed college and above	41 (9.9%)
	Total	416

Religion	Orthodox	203 (48.8%)
	Muslim	38 (9.1%)
	Protestant	163 (39.2%)
	Others^∗^	12 (2.9%)
	Total	416

Ethnicity	Oromo	333 (80.0%)
	Amhara	66 (15.9%)
	Tigre	10 (2.4%)
	Others^∗∗^	7 (1.7%)
	Total	416

Occupation	Farmer	45 (10.8%)
	Merchant	86 (20.7%)
	Employee	141 (33.9%)
	Daily laborer	144 (34.6%)
	Total	416

Languages	Afan Oromo	268 (64.4%)
	Amharic	136 (32.7%)
	Tigrigna	7 (1.7%)
	Others^∗∗∗^	5 (1.2%)
	Total	416

Residence	Urban	233 (56.0%)
	Rural	183 (44.0%)
	Total	416

Climatic condition	Dega	83 (20.0%)
	Weyinadega	308 (74.0%)
	Desert	25 (6.0%)
	Total	416

Farm land	Yes	156 (37.5%)
	No	260 (62.5%)
	Total	416

Irrigation	Yes	88 (21.2%)
	No	328 (78.8%)
	Total	416

Fertilizer	Yes	134 (32.2%)
	No	282 (67.8%)
	Total	416

Monthly income in ETB	≤600	238 (57.2%)
	601-1650	71 (17.1%)
	1651-3200	53 (12.7%)
	3201-5250	43 (10.3%)
	5251-7800	11 (2.6%)
	Total	416

Others^∗^= (Wakefata, Adventist), others^∗∗^= (Gurage, Gumuz), others^∗∗∗^= (sidama).

**Table 2:** Clinical profiles, nutritional, and ART status among people living with HIV/AIDS on follow up at public hospitals of western Ethiopia, 2019 (*N* = 416).

Variables		Category	Frequency (*N*)
WHO clinical stage		1st stage	245 (58.9%)
		2nd stage	58 (13.9%)
		3rd stage	102 (24.5%)
		4th stage	11 (2.6%)
		Total	416

CD4+T cell count		<200 cells/ul	34 (8.2%)
		200-350 cells/ul	114 (27.4%)
		351-500 cells/ul	136 (32.7%)
		>500 cells/ul	132 (31.7%)
		Total	416

Body mass index		≤18	265 (63.7%)
		18-24	125 (30.0%)
		25-29	20 (4.8%)
		≥30	6 (1.4%)
		Total	416

Anemia status		Yes	216 (51.9%)
		No	200 (48.1%)
		Total	416

Cigarette smoking		Yes	254 (61.1%)
		No	162 (38.9%)
		Total	416

Current/past OI in the past six months	No		253 (60.8%)
	Yes	Diarrhea	39 (9.4%)
		TB	16 (3.8%)
		Oral trash	18 (4.3%)
		Pneumocystis Carii	64 (15.4%)
		Others^∗^	26 (6.3%)
		Total	416

ART regimens		(d4T + 3TC + NVP)	73 (17.5%)
		(TDF + 3TC + EFV)	202 (48.6%)
		(AZT + 3TC + NVP)	53 (12.7%)
		(TDF + 3TC + NVP)	66 (15.9%)
		(AZT + 3TC + EFV)	22 (5.3%)
		Total	416

Dietary counseling		Yes	353 (84.9%)
		No	63 (15.1%)
		Total	416

Household dietary diversity		Inadequate dietary diversity	391 (94.0%)
		Adequate dietary diversity	25 (6.0%)
		Total	416

Others^∗^= (skin infection, Asthma).

**Table 3:** Bivariate logistic regression analysis and a test for difference in proportions of factors associated with food insecurity among people living with HIV/AIDS on follow up at public hospitals of western Ethiopia, 2019 (*N* = 416).

Variables	Food insecurity		AOR with 95% CI
Sex	Yes	No	
Male	71 (36.4%)	124 (63.6%)	1.572 (1.036, 2.386)^∗^
Female	59 (26.7%)	162 (73.3%)	1

Marital status			
Married	90 (34.6%)	170 (65.4%)	2.426 (1.236, 4.764)^∗^
Single	22 (36.1%)	39 (63.9%)	2.585 (1.145, 5.837)^∗^
Divorced	6 (21.4%)	22 (78.6%)	1.250 (0.417, 3.746)^∗^
Widowed	12 (17.9%)	55 (82.1%)	1

Educational status			
Illiterate	72 (63.2%)	42 (36.8%)	4.200 (1.530, 11.530)^∗^
Can read and write	73 (68.9%)	33 (31.1%)	3.255 (1.172, 9.042)^∗^
Completed primary	54 (66.7%)	27 (33.3%)	3.600 (1.268, 10.219)^∗^
Completed secondary	51 (68.9%)	23 (31.1%)	3.247 (1.128, 9.345)^∗^
Completed college/university	36 (87.8%)	5 (12.2%)	1

Occupation			
Farmer	39 (86.7%)	6 (13.3%)	1
Merchant	60 (69.8%)	26 (30.2%)	2.817 (1.062, 7.467)^∗^
Employee	97 (68.8%)	44 (31.2%)	2.948 (1.163, 7.476)^∗^
Daily laborer	90 (62.5%)	54 (37.5%)	3.900 (1.549, 9.819)^∗^

Place of residence			
Urban	87 (37.3%)	146 (62.7%)	1.940 (1.259, 2.991)^∗^
Rural	43 (23.5%)	140 (76.5%)	1

Family size			
≤3	83 (35.2%)	153 (64.8%)	2.387 (1.143, 4.987)^∗^
4-5	37 (29.4%)	89 (70.6%)	1.829 (0.833, 4.016)^∗^
>5	10 (18.5%)	44 (81.5%)	1

HIV/AIDS stage			
Stage one	158 (64.5%)	87 (35.5%)	1
Stage two	41 (70.7%)	17 (29.3%)	0.753 (0.404, 1.404)^∗^
Stage three	80 (78.4%)	22 (21.6%)	0.499 (0.291, 0.857)^∗^
Stage four	7 (63.6%)	4 (36.4%)	1.038 (0.296, 3.644)^∗^

Anemia			
Yes	137 (63.4%)	79 (36.6%)	1.685 (1.105, 2.568)^∗^
No	149 (74.5%)	51 (25.5%)	1

Opportunistic infection			
Yes	136 (83.4%)	27 (16.6%)	3.459 (2.134, 5.607)^∗^
No	150 (59.3%)	103 (40.7%)	1

Types of opportunistic diseases			
Diarrhea	32 (82.0%)	7 (18.0%)	5.264 (1.540, 17.993)^∗^
TB	12 (75%)	4 (25%)	1.677 (.392, 7.184)^∗^
Oral trash	13 (72.2%)	5 (27.8%)	2.556 (0.490, 13.329)^∗^
Pneumocystis Carii pneumonia	56 (87.5%)	8 (12.5%)	2.949 (0.605, 14.383)^∗^
Others®	23 (88.5%)	3 (11.5%)	1

Cigarette smoking			
Yes	154 (60.6%)	100 (39.4%)	2.857 (1.786, 4.570)^∗^
No	132 (81.5%)	30 (18.5%)	1

Dietary diversity			
Adequate	12 (48%)	13 (52%)	1
Inadequate	274 (70.1%)	117 (29.9%)	2.537 (1.124, 5.725)^∗^

Others® = (skin infection, Asthma), ^∗^= Significant at *p* < 0.25.

**Table 4:** Multivariable logistic regression analysis of factors associated with food insecurity among people living with HIV/AIDS on follow up public hospitals of western Ethiopia, 2019 (*N* = 416).

Variables	Food insecurity		AOR with 95% CI	AOR with 95% CI
Sex	Yes	No		
Male	71 (36.4%)	124 (63.6%)	1.572 (1.036, 2.386)^∗^	
Female	59 (26.7%)	162 (73.3%)	1	

Marital status				
Married	90 (34.6%)	170 (65.4%)	2.426 (1.236, 4.764)^∗^	3.133 (1.448, 6.782)^∗∗^
Divorced	6 (21.4%)	22 (78.6%)	1.250 (0.417, 3.746)^∗^	1.352 (0.406, 4.502)^∗∗^
Widowed	12 (17.9%)	55 (82.1%)	1	

Educational status				
Illiterate	72 (63.2%)	42 (36.8%)	4.200 (1.530, 11.530)^∗^	5.234 (1.747, 15.686)^∗∗^
Can read and write	73 (68.9%)	33 (31.1%)	3.255 (1.172, 9.042)^∗^	4.330 (1.436, 13.053)^∗∗^
Completed primary	54 (66.7%)	27 (33.3%)	3.600 (1.268, 10.219)^∗^	4.550 (1.472, 14.070)^∗∗^
Completed secondary	51 (68.9%)	23 (31.1%)	3.247 (1.128, 9.345)^∗^	3.400 (1.102, 10.493)^∗∗^
Completed college/university	36 (87.8%)	5 (12.2%)	1	

Occupation				
Farmer	39 (86.7%)	6 (13.3%)	1	
Merchant	60 (69.8%)	26 (30.2%)	2.817 (1.062, 7.467)^∗^	
Employee	97 (68.8%)	44 (31.2%)	2.948 (1.163, 7.476)^∗^	
Daily laborer	90 (62.5%)	54 (37.5%)	3.900 (1.549, 9.819)^∗^	

Place of residence				
Urban	87 (37.3%)	146 (62.7%)	1.940 (1.259, 2.991)^∗^	
Rural	43 (23.5%)	140 (76.5%)	1	

Family size				
≤3	83 (35.2%)	153 (64.8%)	2.387 (1.143, 4.987)^∗^	
4-5	37 (29.4%)	89 (70.6%)	1.829 (0.833, 4.016)^∗^	
>5	10 (18.5%)	44 (81.5%)	1	

HIV/AIDS stage				
Stage one	158 (64.5)	87 (35.5%)	1	
Stage two	41 (70.7%)	17 (29.3%)	753 (0.404, 1.404)^∗^	
Stage three	80 (78.4%)	22 (21.6%)	499 (0.291, 0.857)^∗^	
Stage four	7 (63.6%)	4 (36.4%)	1.038 (0.296, 3.644)^∗^	

Anemia				
Yes	137 (63.4)	79 (36.6%)	1.685 (1.105,2.568)^∗^	2.650 (1.563, 4.493)^∗∗^
No	149 (74.5)	51 (25.5%)	1	1

Opportunistic infection				
Yes	136 (83.4%)	27 (16.6%)	3.459 (2.134, 5.607)^∗^	3.108 (1.755, 5.505)^∗∗^
No	150 (59.3%)	103 (40.7%)	1	1

Types of opportunistic diseases				
Diarrhea	32 (82.0%)	7 (18.0%)	5.264 (1.540,17.993)^∗^	
TB	12 (75%)	4 (25%)	1.677 (0.392,7.184)^∗^	
Oral trash	13 (72.2%)	5 (27.8%)	2.556 (0.490, 13.329)^∗^	
Pneumocystis Carii pneumonia	56 (87.5%)	8 (12.5%)	2.949 (0.605, 14.383)^∗^	
Others®	23 (88.5%)	3 (11.5%)	1	

Cigarette smoking				
Yes	154 (60.6%)	100 (39.4%)	2.857 (1.786,4.570)^∗^	3.577 (2.104, 6.081)^∗∗^
No	132 (81.5%)	30 (18.5%)	1	1

Dietary diversity				
Adequate	12 (48%)	13 (52%)	1	1
Inadequate	274 (70.1%)	117 (29.9%)	2.537 (1.124, 5.725)^∗^	2.870 (1.088, 7.569)^∗∗^

^∗^= significant at *p* < 0.25^∗∗^= significant at *p* < 0.05.

## Data Availability

The data used during this study are available from the corresponding author on reasonable request.

## Abbreviations

AIDS: Acquired immunodeficiency syndrome
ART: Antiretroviral therapy
BSC: Bachelor science
FAO: Food and agricultural organization
FANTA: Food and nutrition technical assistance
FFQs: Food frequency questionnaires
HAART: Highly active antiretroviral therapy
HFIAS: Household food insecurity access scale
HID: Health information dissemination
HIV: Human immuno virus
OSSA: Organization for Social Service Association
PLWHA: People living with HIV AIDS
SSA: Sub-Saharan Africa
WHO: World Health Organization.
